# Mild Behavioral Impairment Is Associated With Atrophy of Entorhinal Cortex and Hippocampus in a Memory Clinic Cohort

**DOI:** 10.3389/fnagi.2021.643271

**Published:** 2021-05-24

**Authors:** Veronika Matuskova, Zahinoor Ismail, Tomas Nikolai, Hana Markova, Katerina Cechova, Zuzana Nedelska, Jan Laczó, Meng Wang, Jakub Hort, Martin Vyhnalek

**Affiliations:** ^1^Memory Clinic, Department of Neurology, Second Faculty of Medicine, Charles University and Motol University Hospital, Prague, Czechia; ^2^International Clinical Research Center, St. Anne’s University Hospital Brno, Brno, Czechia; ^3^Department of Psychiatry, Cumming School of Medicine, Calgary, AB, Canada; ^4^Department of Clinical Neurosciences, Cumming School of Medicine, Calgary, AB, Canada; ^5^Department of Community Health Sciences, Cumming School of Medicine, Calgary, AB, Canada; ^6^Hotchkiss Brain Institute and O’Brien Institute for Public Health, University of Calgary, Calgary, AB, Canada

**Keywords:** entorhinal cortex, hippocampus, mild behavioral impairment-checklist, mild cognitive impairment, neuropsychiatric symptoms, subjective cognitive decline, magnetic resonance imaging

## Abstract

**Objectives:**

Mild behavioral impairment (MBI) is a syndrome describing late-onset persistent neuropsychiatric symptoms (NPS) in non-demented older adults. Few studies to date have investigated the associations of MBI with structural brain changes. Our aim was to explore structural correlates of NPS in a non-demented memory clinic sample using the Mild Behavioral Impairment Checklist (MBI-C) that has been developed to measure MBI.

**Methods:**

One hundred sixteen non-demented older adults from the Czech Brain Aging Study with subjective cognitive concerns were classified as subjective cognitive decline (*n* = 37) or mild cognitive impairment (*n* = 79). Participants underwent neurological and neuropsychological examinations and brain magnetic resonance imaging (MRI) (1.5 T). The Czech version of the MBI-C was administered to participants’ informants. Five *a priori* selected brain regions were measured, namely, thicknesses of the orbitofrontal cortex (OFC), anterior cingulate cortex (ACC), posterior cingulate cortex (PCC), and entorhinal cortex (ERC) and volume of the hippocampus (HV), and correlated with MBI-C total and domain scores.

**Results:**

Entorhinal cortex was associated with MBI-C total score (*r*_*S*_ = −0.368, *p* < 0.001) and with impulse dyscontrol score (*r*_*S*_ = −0.284, *p* = 0.002). HV was associated with decreased motivation (*r*_*S*_ = −0.248, *p* = 0.008) and impulse dyscontrol score (*r*_*S*_ = −0.240, *p* = 0.011).

**Conclusion:**

Neuropsychiatric symptoms, particularly in the MBI impulse dyscontrol and motivation domains, are associated with medial temporal lobe atrophy in a clinical cohort of non-demented older adults. This study supports earlier involvement of temporal rather than frontal regions in NPS manifestation. Since these regions are typically affected early in the course of Alzheimer’s disease (AD), the MBI-C may potentially help further identify individuals at-risk of developing AD dementia.

## Introduction

Neuropsychiatric symptoms (NPS) are a common feature in early stages of various neurodegenerative diseases (Desmarais et al., 2018; Fischer and Agüera-Ortiz, 2018; Ismail et al., 2018; Sherman et al., 2018; Bateman et al., 2020) and can precede the onset of dementia by several years (Singh-Manoux et al., 2017; Tapiainen et al., 2017). NPS may emerge before detectable cognitive decline and are associated with a higher risk of clinical progression to mild cognitive impairment (MCI) or dementia (Sugarman et al., 2018; Wise et al., 2019). There is compelling evidence linking various NPS, such as depression, anxiety, apathy, agitation, and irritability, to accumulation of beta-amyloid, a hallmark of Alzheimer’s disease (AD) (Bensamoun et al., 2016; Ng et al., 2017; Gatchel et al., 2019; Goukasian et al., 2019; Johansson et al., 2020), but these symptoms can also occur in physiological aging (Bunce et al., 2012) or can be related to other pathologies, such as cerebrovascular disease (Tiel et al., 2015). Thus, their clinical interpretation is often challenging.

To improve the detection of early stages of neurodegenerative diseases, a new neurobehavioral syndrome, named mild behavioral impairment (MBI), has been recently proposed by the Alzheimer’s Association Working Group (Ismail et al., 2016). MBI describes new-onset and persistent NPS in non-demented older adults (reflecting a change from baseline patterns of behavior), as an at-risk state for incident cognitive decline and dementia. MBI can emerge not only in persons with MCI but also in cognitively normal (CN) older adults, in whom MBI is associated with a greater risk of incident cognitive decline and dementia (Taragano et al., 2018; Matsuoka et al., 2019; Ismail et al., 2021). MBI (assessed with the Neuropsychiatric Inventory) was highly prevalent in both clinical and community-based cohorts of non-demented older adults (Mortby et al., 2018; Sheikh et al., 2018).

To verify the presence of MBI and to address the need for a more sensitive and specific NPS scale in preclinical and prodromal stages of neurodegenerative diseases, a new instrument, the Mild Behavioral Impairment Checklist (MBI-C) (Ismail et al., 2017a), has been developed. The MBI-C serves as a global and domain-specific NPS measure including early symptom presentations. Its factor structure has been validated in older adults with normal cognition (Creese et al., 2020), as have its psychometric properties to capture MBI in subjective cognitive decline (SCD) (Mallo et al., 2018b) and MCI (Mallo et al., 2018a) populations. Thus, the MBI-C may provide a more precise alternative to routinely used NPS measures that have been mostly developed to assess NPS in the dementia stage. A large population-based study of non-demented older adults using the MBI-C reported at least one symptom in more than half of the participants, with affective and impulse dyscontrol symptoms being the most commonly reported (Creese et al., 2019).

Recent studies have demonstrated a link between higher MBI-C score and increased beta-amyloid pathology (a higher global and regional beta-amyloid PET uptake) in CN older adults (Lussier et al., 2020). The presence of MBI in non-demented ADNI participants predicted a higher increase in a plasma neurofilament light protein over 2 years compared to the participants without MBI, bringing evidence for the link between MBI and subsequent neurodegeneration (Naude et al., 2020). Furthermore, MBI has demonstrated an association with AD risk genes (Andrews et al., 2018; Creese et al., 2021). Thus, it seems that MBI also identifies a potential at-risk group for incident cognitive decline and AD dementia. However, studies that explored structural neuroimaging correlates of MBI in older adults at-risk for AD dementia are scarce.

Using the informant version of the MBI-C, we aimed to examine the associations between regional brain atrophy on magnetic resonance imaging (MRI) and NPS severity in a memory clinic cohort of clinically and neuropsychologically well-defined non-demented older adults. Five brain regions were selected based on their previously reported associations with NPS [thicknesses of the orbitofrontal cortex and anterior cingulate cortex (Rosenberg et al., 2015; Boublay et al., 2016)] and with early AD pathology [posterior cingulate cortex, entorhinal cortex, and hippocampal volume (Braak et al., 2006)]. To measure NPS severity, MBI-C total score and five MBI-C domain scores were used. We hypothesized that in this cohort of individuals at risk of developing AD dementia, (1) participants with higher MBI burden would have more pronounced atrophy, above and beyond demographics (age, sex, and years of education) and global cognitive status; and (2) across the MBI domains, more pronounced regional atrophy would be associated particularly with more severe affective, decreased motivation, and impulse dyscontrol symptoms, which are the NPS known to be present in early stages of AD (Bensamoun et al., 2016; Ng et al., 2017; Gatchel et al., 2019; Johansson et al., 2020).

## Materials and Methods

### Participants

A total of 116 participants with subjective cognitive concerns were included in this study. Participants were recruited from the Czech Brain Aging Study (CBAS), an ongoing longitudinal memory clinic-based study aimed at detecting early changes associated with pathological brain aging (Sheardova et al., 2019). Participants with subjectively perceived cognitive complaints were referred to our memory clinic by general practitioners or other specialists. All participants underwent standard neurological and laboratory evaluations, comprehensive neuropsychological examination, and 1.5-T brain magnetic resonance imaging (MRI) within 3 months from the initial visit. Based on the neuropsychological examination, the participants were classified as either SCD or MCI. SCD (*n* = 37) was diagnosed using the SCD-Initiative Workgroup criteria (Jessen et al., 2014) and included persons with (1) subjectively perceived cognitive decline compared to a previously normal status, unrelated to an acute event, and (2) no objective cognitive impairment in the neuropsychological assessment based on age-, gender-, and education-adjusted norms. Subjective cognitive complaints were assessed by an experienced neuropsychologist in a semi-structured interview. MCI (*n* = 79) was diagnosed according to the NIA-AA 2011 criteria (Albert et al., 2011) and included persons with the following: (1) subjectively perceived cognitive decline compared to a previously normal status; (2) neuropsychologically confirmed objective cognitive impairment below 1.5 SD on at least two tests within a domain in at least one of five established cognitive domains; (3) preservation of independence in functional abilities; and (4) the absence of dementia. Both amnestic (*n* = 66; aMCI) and non-amnestic (*n* = 13; naMCI) MCI were included. All the diagnoses were made by consensus involving cognitive neurologists and neuropsychologists. The exclusion criteria were as follows: (1) a diagnosis of dementia; (2) the presence of other neurologic or psychiatric diseases [e.g., Parkinson’s disease, traumatic brain injury, stroke, alcohol or substance abuse, severe brain vascular burden (Fazekas scale ≥ 2 on MRI), current major psychiatric disorder, or a history of major psychiatric disorder as confirmed by clinical interviews]. The demographic characteristics of the participants are presented in Table 1.

**TABLE 1:** Demographic, neuropsychological and volumetric characteristics of the participants.

Characteristics	SCD *n* = 37 *M* ± SD	MCI *n* = 79 *M* ± SD	All *n* = 116 *M* ± SD
Age^*a*^	66.37 ± 6.71	71.05 ± 8.41**	69.56 ± 8.18
Female, *n* (%)^*b*^	22 (59.5)	35 (44.3)	57 (49)
MMSE, score^*a*^	29.30 ± 0.88	26.82 ± 2.51**	27.61 ± 2.42
Education^*a*^	16.55 ± 3.21	14.82 ± 3.16**	15.38 ± 3.26
RAVLT 1-5, score^*a*^	55.41 ± 7.44	35.05 ± 11.60**	41.78 ± 14.14
RAVLT delayed recall, score^*a*^	12.03 ± 2.29	4.69 ± 3.73**	7.12 ± 4.79
LM delayed recall, score^*a*^	15.46 ± 3.91	8.08 ± 5.52**	10.43 ± 6.12
WAIS-III Digit span, score^*a*^	16.27 ± 3.23	13.67 ± 3.71**	14.50 ± 3.75
TMT A, time to completion (s)^*a*^	36.35 ± 7.11	55.72 ± 29.53**	49.49 ± 26.23
TMT B, time to completion (s)^*a*^	76.30 ± 21.25	166.64 ± 81.63**	137.58 ± 80.25
BNT-30, mistakes after a semantic cue^*a*^	1.62 ± 1.79	5.00 ± 3.91**	3.92 ± 3.73
C-VF animals, score^*a*^	26.43 ± 5.46	18.25 ± 5.38**	20.86 ± 6.60
P-VF, score^*a*^	52.08 ± 11.79	36.68 ± 12.98**	41.59 ± 14.48
PST—colors, time to completion (s)^*a*^	28.33 ± 6.68	42.64 ± 18.17**	38.00 ± 16.78
ROCF copy, score^*a*^	31.24 ± 3.11	26.70 ± 5.87**	28.16 ± 5.55
ROCF recall, score^*a*^	19.35 ± 6.38	9.40 ± 6.17**	12.60 ± 7.77
ERC, mm^*a*^	3.30 ± 0.43	2.91 ± 0.46**	3.03 ± 0.48
HV, mm^3*a*^	3,935.83 ± 434.98	3,381.10 ± 607.54**	3,558.04 ± 614.01
ACC, mm^*a*^	2.74 ± 0.18	2.73 ± 0.21	2.73 ± 0.20
PCC, mm^*a*^	2.37 ± 0.11	2.33 ± 0.21	2.33 ± 0.18
OFC, mm^*a*^	2.52 ± 0.12	2.40 ± 0.21**	2.44 ± 0.19
AMG, mm^3*a*^	1,407.97 ± 182.21	1,281.90 ± 232.91*	1,322.12 ± 225.10

*Difference from SCD group at *p* < 0.05; **difference from SCD group at *p* < 0.01. ^*a*^*t*-test; ^*b*^chi-square. SCD, subjective cognitive decline; MCI, mild cognitive impairment; MMSE, Mini-Mental State Examination; LM, logical memory; RAVLT, Rey Auditory Verbal Learning Test; TMT, Trial Making Test; BNT (30), Boston Naming Test—30-item version; C-VF, category verbal fluency; P-VF, phonemic verbal fluency—letters N, K, and P; PST, Prague Stroop Test; ROCF, Rey–Osterrieth complex figure; ERC, entorhinal cortex thickness; HV, hippocampal volume; ACC, anterior cingulate cortex thickness; PCC, posterior cingulate cortex thickness; OFC, orbitofrontal cortex thickness; AMG, amygdala volume; *M*, mean; SD, standard deviation.

All participants provided written informed consent according to the Declaration of Helsinki and the study was approved by the Ethics Committee of Motol University Hospital.

### Neuropsychological Assessment

The neuropsychological battery included the Mini-Mental State Examination (MMSE) as a screening of global cognitive function and the following tests to assess five (Albert et al., 2011) cognitive domains: (1) memory by the Rey Auditory Verbal Learning Test (RAVLT), Logical Memory from the Wechsler Memory Scale-Third Edition (LM), and Rey–Osterrieth Complex Figure Test (ROCFT recall after 3 min); (2) executive function by the Trail Making Test B (TMT B), phonemic verbal fluency—letters N, K, and P (P-VF) and Prague Stroop test—colors (PST); (3) language by the Boston Naming Test 30-item version (BNT) and category verbal fluency—animals (C-VF); (4) attention and working memory by the Trial Making Test A (TMT A) and Digit Span Forward and Backward (DS) from the Wechsler Adult Intelligence Scale-Third Edition; and (5) visuospatial function by the Rey–Osterrieth Complex Figure Test (ROCFT copy) (Nikolai et al., 2018). All scores are presented in Table 1.

### Neuropsychiatric Assessment

The Czech version of the MBI-C (Matuskova et al., 2020) was completed by a participant’s close informant (a partner, a descendant, or another relative). The MBI-C is a newly developed instrument specifically designed to assess neuropsychiatric symptoms before the onset of dementia (Ismail et al., 2017a). It comprises of 34 items evaluating five behavioral domains in line with the recently proposed MBI diagnostic criteria (Ismail et al., 2016): (1) decreased motivation (six questions assessing cognitive, emotional, and behavioral apathy); (2) affective dysregulation (six questions evaluating depressive and anxiety symptoms); (3) impulse dyscontrol (12 questions assessing symptoms of agitation, aggression, impulsivity, and abnormal reward salience); (4) social inappropriateness (five questions describing tact, empathy, and sensitivity); and (5) abnormal perception and thought content (five questions regarding suspiciousness, grandiosity, and hallucinations). Each question requires an answer regarding presence (yes/no) and severity of the symptoms (1—mild, 2—moderate, 3—severe). The symptoms should represent a change from the person’s usual behavior and persist over the last 6 months. The total score as well as five domain scores were calculated as a sum of the corresponding item severity ratings resulting in the MBI-C total score (0–102), decreased motivation score (0–18), affective dysregulation score (0–18), impulse dyscontrol score (0–36), social inappropriateness score (0–15), and abnormal perception and thought content score (0–15). *Z* scores were calculated for the MBI-C total score and all the domain scores for the whole cohort. Participants with four or more missing items on the MBI-C were excluded. In case of three or fewer missing items, both total and domain scores were calculated without these items. All scores are presented in Table 2.

**TABLE 2:** MBI-C scores of the participants.

MBI-C, score (range)^*a*^	SCD *n* = 37 *M* ± SD	MCI *n* = 79 *M* ± SD	All *n* = 116 *M* ± SD
Total (0−102)	2.65 ± 4.27 (0−18)	5.38 ± 5.66 (0−27)**	4.51 ± 5.39 (0−27)
Decreased motivation (0−18)	0.46 ± 1.12 (0−5)	1.48 ± 2.01 (0−8)**	1.16 ± 1.83 (0−8)
Affective dysregulation (0−18)	0.97 ± 1.57 (0−7)	1.75 ± 2.14 (0−9)*	1.50 ± 2.00 (0−9)
Impulse dyscontrol (0−36)	1.00 ± 2.15 (0−9)	1.77 ± 2.28 (0−9)*	1.53 ± 2.26 (0−9)
Social inappropriateness (0−15)	0.19 ± 0.52 (0−2)	0.25 ± 0.59 (0−3)	0.23 ± 0.57 (0−3)
Abnormal perception/thought (0−15)	0.03 ± 0.16 (0−1)	0.11 ± 0.39 (0−2)	0.09 ± 0.34 (0−2)

*Difference from SCD group at *p* < 0.05; **difference from SCD group at *p* < 0.01. ^*a*^Mann–Whitney *U* test.SCD, subjective cognitive decline; MCI, mild cognitive impairment; MBI-C, Mild behavioral impairment checklist; *M*, mean; SD, standard deviation.

### MRI Acquisition and Processing

Brain scans were performed on a 1.5-T scanner (Siemens AG, Erlangen, Germany) using the T1-weighted three-dimensional high-resolution magnetization-prepared rapid gradient-echo (MP-RAGE) sequence with TR/TE/TI = 2,000/3.08/1,100 ms, flip angle 15°, 192 continuous partitions, slice thickness = 1.0 mm, and in-plane resolution = 1 mm. Scans were visually checked by an image analyst to ensure appropriate data quality and by a neuroradiologist to exclude participants with a major pathology interfering with cognitive functioning such as cortical infarctions, tumor, subdural hematoma, and hydrocephalus.

Volumetric segmentation and cortical reconstruction were performed using the FreeSurfer image analysis software (version 5.3), which is documented and freely available^1^. FreeSurfer is a popular and widely used algorithm and has been described in detail and well documented in prior publications (Fischl and Dale, 2000; Fischl et al., 2002, 2004a,b; Ségonne et al., 2004; Han et al., 2006; Jovicich et al., 2006; Reuter et al., 2010). Cortical thicknesses in four regions were selected based on previously reported associations with NPS (orbitofrontal cortex, OFC; anterior cingulate cortex, ACC) (Rosenberg et al., 2015; Boublay et al., 2016) or with early AD pathology (posterior cingulate cortex, PCC; entorhinal cortex, ERC) (Braak et al., 2006). Regional thickness from the left and right hemispheres was averaged. We also included hippocampal volumes (HV), adjusted for the total estimated intracranial volume (eTIV) using the proportion method (O’Brien et al., 2011), and right and left HV were averaged. Volumetric characteristics are presented in Table 1. Subsequently, we performed an exploratory analysis with amygdala volume, adjusted for the total estimated intracranial volume (eTIV). These results are presented in the Supplementary Material (part 2).

### Statistical Analysis

To explore the differences between SCD and MCI groups, we performed *t*-tests for normally distributed data. Due to a non-normal distribution, Mann–Whitney *U* tests were used to compare MBI total and domain scores between the groups. Chi-square test was used to examine differences in sex proportion. All subsequent analyses were performed within the whole sample (SCD + MCI). Because of a non-normal, right-skewed distribution of the MBI-C scores, the associations of the MBI-C total score and five domain scores with the ROIs were assessed using covariate-adjusted Spearman rank correlations. In the first model, the correlations were adjusted for age, sex, and years of education. In the second model, we included additional adjustment for MMSE. Furthermore, we performed complementary analyses excluding naMCI participants (resulting in *n* = 103). The analyses were corrected for multiple comparisons using the Holm–Bonferroni correction (corrected for the number of tests performed per each MBI score). All the analyses were performed using SPSS version 20.

## Results

Participants’ demographic, neuropsychological, and MRI characteristics are presented in Table 1. MBI-C scores are presented in Table 2. Most of the informants that completed the MBI-C were participants’ spouses/partners (70%) or descendants (22%). Overall, 70% of the cohort reported at least one symptom (scored ≥ 1) in the MBI-C (54% of SCD and 78.5% of MCI). Symptoms of affective dysregulation and impulse dyscontrol were the most common, while abnormal perception/thought content were the least common (see Table 2).

In the entire cohort, MBI-C total score was weakly associated with ERC thickness, controlling for age, sex, and education. Within MBI-C domains, impulse dyscontrol score was moderately associated with ERC thickness and weakly associated with HV, the former remaining significant after additional controlling for MMSE. Decreased motivation score was weakly associated with HV; however, this association disappeared after additional controlling for MMSE.

Moreover, HV was weakly associated with MBI-C total score, and OFC thickness was weakly associated with MBI-C total score, impulse dyscontrol, and decreased motivation; the two former associations survived additional controlling for MMSE. However, all these associations disappeared after correcting for multiple comparisons. No other associations were observed. All results are presented in Table 3. Excluding naMCI participants (*n* = 13) from the analyses did not substantially change the results (see part 1 in the Supplementary Material).

**TABLE 3:** Associations of cortical thickness and volume measures with MBI-C total and domain scores.

	Spearman *r*_*S*_, *p* value adjusted for age, sex, and education						Spearman *r*_*S*_, *p* value adjusted for age, sex, education, and MMSE					
	MBI-C total score	MBI-C Motivation	MBI-C Affectivity	MBI-C Impulse dyscontrol	MBI-C Social	MBI-C Perception/thought	MBI-C total score	MBI-C Motivation	MBI-C Affectivity	MBI-C Impulse dyscontrol	MBI-C Social	MBI-C Perception/thought
ERC†	−0.284, 0.002*	−0.138, 0.146	−0.154, 0.103	−0.368, < 0.001*	−0.130, 0.169	−0.125, 0.187	−0.238, 0.012	−0.087, 0.361	−0.110, 0.248	−0.337, < 0.001*	−0.137, 0.151	−0.132, 0.165
HV††	−0.225, 0.016	−0.248, 0.008*	−0.060, 0.527	−0.240, 0.011*	−0.182, 0.053	0.031, 0.744	−0.168, 0.077	−0.199, 0.035	−0.002, 0.985	−0.196, 0.038	−0.195, 0.040	0.033, 0.731
ACC†	−0.018, 0.851	0.065, 0.497	−0.011, 0.907	−0.051, 0.589	−0.171, 0.070	−0.123, 0.196	−0.023, 0.813	0.062, 0.518	−0.015, 0.877	−0.056, 0.560	−0.171, 0.071	−0.123, 0.198
PCC†	−0.058, 0.544	−0.013, 0.889	−0.014, 0.887	−0.150, 0.113	−0.078, 0.409	−0.122, 0.197	−0.051, 0.590	−0.007, 0.942	−0.008, 0.935	−0.146, 0.125	−0.078, 0.411	−0.122, 0.199
OFC†	−0.214, 0.023	−0.201, 0.033	−0,127, 0.182	−0.206, 0.028	−0.125, 0.186	−0.099, 0.295	−0.197, 0.038	−0.185, 0.051	−0.110, 0.247	−0.191, 0.043	−0.126, 0.186	−0.100, 0.294

^†^Cortical thickness (averaged between left and right hemisphere); ^†⁣†^volume (averaged between left and right hemisphere) adjusted for eTIV (proportion method); *significant after applying Holm–Bonferroni correction for multiple comparisons. MBI-C, mild behavioral impairment checklist; ERC, entorhinal cortex thickness; HV, hippocampal volume (eTIV); ACC, anterior cingulate cortex thickness; PCC, posterior cingulate cortex thickness; OFC, orbitofrontal cortex thickness.

Following our finding that MBI was mostly associated with the two medial temporal lobe (MTL) structures, we wanted to further explore the associations with the amygdala, a key MTL structure involved in neuropsychiatric manifestations. In the exploratory analysis, we found that the amygdala was not associated with any of the MBI-C scores (see part 2 of the Supplementary Material).

## Discussion

To the best of our knowledge, this is the first study using the MBI-C to examine the relationships between regional brain atrophy and NPS in a well-characterized sample of non-demented memory clinic participants enrolled in the Czech Brain Aging Study. We assessed the association between atrophy in five *a priori* selected brain regions and MBI-C severity. We found that MBI-C total score as well as impulse dyscontrol and decreased motivation domain scores were associated with atrophy in two medial temporal lobe (MTL) regions, i.e., the ERC and hippocampus.

Medial temporal lobe regions (known to be affected early in AD) have traditionally been associated with cognitive functions, especially episodic memory and spatial navigation (Braak et al., 2006; Laczó et al., 2017; Berron et al., 2020). However, evidence suggests that their anterior parts belong to a distinct functional subsystem (Ranganath and Ritchey, 2012), which is involved in the regulation of motivational and emotional behavior (Fanselow and Dong, 2010). Disruption of this integrity may therefore also manifest with various NPS. This is supported by several recent studies. It has been shown that accumulation of tau pathology in the ERC and inferior temporal lobe (Gatchel et al., 2017b) and lower HV (Donovan et al., 2015) are associated with depressive symptoms in CN older adults. The hippocampus is also among the structures associated with apathy in non-demented older adults (Johansson et al., 2020) and with agitation in MCI and AD (Trzepacz et al., 2013). Our findings are therefore consistent with prior reports describing MBI as a manifestation of neurodegeneration (Ismail et al., 2016) and, in some, a manifestation of early AD. The latter is also supported by research linking MBI to AD pathology in cognitively normal older adults (Lussier et al., 2020; Johansson et al., 2021).

We found impulse dyscontrol domain to be the most strongly associated with MTL atrophy. This complies with a very recent finding that impulse dyscontrol was associated with tau deposition in the ERC and hippocampus in preclinical AD (Johansson et al., 2021). Also, an ADNI machine learning study found that baseline presence of these symptoms was an important input feature for cognitive category classification (CN, MCI, and dementia) at 40 months (Gill et al., 2020). In a mixed cohort of CN, MCI, and AD dementia, impulse dyscontrol was associated with lower white matter integrity and lower thickness of the parahippocampal gyrus (Gill et al., 2021). Impulse dyscontrol is a heterogeneous domain, including symptoms such as agitation, disinhibition, abnormal reward salience, or impaired oral intake (Ismail et al., 2016; Saari et al., 2021). In our study, the items mostly endorsed by the informants included agitation, argumentativeness, impatience, and rigidity (items 1, 2, 5, and 7), which were also found to be the most prevalent in a similar cohort (Saari et al., 2021) and were previously designated as an “agitation” cluster (Creese et al., 2019). Neurodegenerative changes in MTL may thus manifest as this cluster of agitation symptoms, possibly as a result of impaired inhibition of emotional responses in stressful interpersonal situations (Sturm et al., 2013). In line with a recent network analysis of impulse dyscontrol domain in SCD and MCI, they may represent a cluster of impulsive behaviors observed in social settings rather than generalized impulsivity or compulsive behavior (Saari et al., 2021).

Lower HV in our study was also associated with apathy (decreased motivation domain), although to a lesser extent. This is in accordance with apathy being consistently reported as one of the most prevalent symptoms in non-demented older adults and associated with AD pathology (Sherman et al., 2018; Johansson et al., 2020).

Medial temporal lobe also comprises the amygdala, a key structure that regulates emotional behavior. It has been suggested that in early AD, amygdala atrophy is comparable to hippocampal atrophy (Poulin et al., 2011). We thus conducted an exploratory analysis to see whether there is an association between MBI-C scores and amygdala volume. None of the MBI-C scores were associated with amygdala volume, which is in line with a previous study (Poulin et al., 2011) and suggests that, from the main MTL structures explored here, MBI symptoms are specific to ERC and hippocampal atrophy.

Interestingly, we found no associations between atrophy in our selected regions and symptoms of depression and anxiety (affective dysregulation domain) although these were among the most prevalent in our cohort, are among the most commonly reported in non-demented older adults (Ismail et al., 2018), and have been associated with an increased risk of progression to AD dementia (Pietrzak et al., 2015; Gallagher et al., 2018; Gatchel et al., 2019). There may be several explanations for these findings. First, depressive and anxiety symptoms may not be specific to neurodegenerative changes in regions included here. Instead, they may be caused by other pathological changes, including serotonin or noradrenaline deficiency (Šimić et al., 2017) or comorbid white matter pathology (Puzo et al., 2019). Furthermore, there is substantial overlap between key symptoms of depression and apathy, and they may often co-occur (Nobis and Husain, 2018). In a study in CN older adults using GDS-derived clusters of symptoms, only apathy/anhedonia but not anxiety/concentration were associated with both reduced HV volume and posterior cortical hypometabolism (Donovan et al., 2015). Another longitudinal study of older adults reported that anhedonia, but not dysphoria, is a risk factor for conversion to dementia (Lee et al., 2019). In the MBI-C, these symptoms are also part of decreased motivation domain. The use of different NPS instruments may thus be another possible reason for these differences. Altogether, previous findings along with ours may thus not be conflicting, but rather describe a broader array of symptoms that emerge early in the course of AD.

We found no associations between PCC thickness and MBI-C scores, although PCC is involved in NPS pathophysiology in early AD. PCC hypometabolism was associated with higher apathy scores both cross-sectionally and over time across the AD clinical spectrum (Gatchel et al., 2017a). Individuals with preclinical AD with more severe NPS displayed metabolic dysfunction in PCC at baseline and 2-year follow-up (Ng et al., 2017). The discrepancy between positive findings from metabolic studies and the absence of a relationship in our study could be explained by the fact that, in AD, metabolic changes in this region precede atrophy (Rodriguez-Oroz et al., 2015).

We also did not observe any associations between ACC or OFC thickness and MBI-C scores. This is in contrast with previous research on various NPS that have identified consistent abnormalities mainly within ACC and frontal regions (Rosenberg et al., 2015; Boublay et al., 2016). There may be several explanations for this. First, our study involved persons in early stages of cognitive impairment (i.e., mean MMSE in MCI group was 26.82 ± 2.51) compared to previous research mostly focusing on persons with AD dementia (Rosenberg et al., 2015; Boublay et al., 2016). This is also supported by no significant differences in ACC and OFC thicknesses between our SCD and MCI groups (see Table 1). Similarly, previous studies exploring structural correlates of apathy in CN and MCI individuals also reported no (Guercio et al., 2015) or weaker (Johansson et al., 2020) associations with atrophy in ACC or frontal regions (compared to temporal regions) and concluded that these associations may only be evident in later stages of AD. Our findings are consistent with this hypothesis. Furthermore, inconsistent findings may stem from different study cohorts. In non-demented older adults referred for progressive behavioral symptoms, MBI was associated with isolated frontal atrophy and a higher risk of progression to behavioral variant of frontotemporal dementia at 4 years (Orso et al., 2020). Cognitive complaints were an exclusion criterion in that study, as opposed to our study, in which they represented the main reason for referral to our clinic. This may have accounted for different regional atrophy being associated with MBI, possibly resulting from a distinct underlying pathology. Finally, we found several associations with OFC that disappeared after correction for multiple comparisons. Together with the fact that OFC is heavily connected with the hippocampus and other MTL structures and their interaction is important for selecting appropriate behavioral responses based on changing social cues (Catenoix et al., 2005; Ranganath and Ritchey, 2012; Ross et al., 2013), we may hypothesize that MBI-C symptoms may also reflect impaired MTL-OFC interaction.

We acknowledge several limitations to our study. First, a memory clinic setting limits the generalizability of our results to a general population, as neuropsychiatric symptoms are more frequent in clinical versus community samples (Ismail et al., 2017b). Another issue to consider is the lack of beta-amyloid and tau biomarkers, which limits our ability to attribute the structural changes we observed truly to AD pathology, notwithstanding the fact that all participants presented to clinic with cognitive concerns. Furthermore, some MBI-C domains (social inappropriateness and abnormal perception/thought) had very low mean scores and a limited range of scores. Thus, the opportunity to detect associations may be low. Finally, it would be pertinent to examine MBI symptoms also in SCD and MCI groups separately since detecting MBI may be particularly useful in persons with no cognitive impairment in terms of identifying at-risk individuals (Ismail et al., 2020). The modest sample size in our study did not allow us to perform the analysis in these subgroups, and we addressed this issue by controlling the analyses for age and global cognition, represented by MMSE. On the other hand, even though we recognize these two separate groups in our sample, we consider them a cognitive continuum of non-demented older adults presenting with subjective cognitive concerns that urged them to seek medical help. They underwent the same recruiting process as they were recruited from the same CBAS cohort. Including both groups also improved our ability to detect associations with volumetric measures of brain atrophy, which may be more challenging to capture in SCD only.

## Conclusion

In summary, using the MBI-C, designed to assess a broad spectrum of early and persistent NPS in predementia stages, we found symptoms of apathy and impulse dyscontrol to be associated with medial temporal lobe atrophy, but not with frontal lobe atrophy. This study suggests that there is a wider group of neuropsychiatric symptoms emerging early in the course of neurodegeneration and supports earlier involvement of temporal rather than frontal regions in their manifestation. Our findings also support MBI-C as a valid tool for detecting NPS in a clinical cohort of non-demented older adults, which may be potentially useful in further detection of individuals at-risk of developing AD dementia. A research implementing longitudinal study design and AD biomarkers is ongoing.

## Data Availability Statement

The datasets presented in this article are not readily available because of the policy of the Czech Brain Aging Study (CBAS), which allows sharing of the data only after previous approval. Requests to access the datasets should be directed to MV, martin.vyhnalek@fnmotol.cz.

## Ethics Statement

The studies involving human participants were reviewed and approved by Ethics Committee of Second Faculty of Medicine, Charles University and Motol University Hospital. The participants provided their written informed consent to participate in this study.

## Author Contributions

MV, VM, and ZI participated in the design of the study and data interpretation and drafted the manuscript. VM, HM, KC, TN, MV, JL, and JH were involved in data acquisition and interpretation. ZN was involved in MRI data acquisition and processing. VM and MW performed the statistical analyses. All authors read and approved the final manuscript.

## Conflict of Interest

The authors declare that the research was conducted in the absence of any commercial or financial relationships that could be construed as a potential conflict of interest.

## Footnotes

1
http://surfer.nmr.mgh.harvard.edu/
