# Corrigendum: Mild Behavioral Impairment Is Associated With Atrophy of Entorhinal Cortex and Hippocampus in a Memory Clinic Cohort

**DOI:** 10.3389/fnagi.2022.884077

**Published:** 2022-04-28

**Authors:** Veronika Matuskova, Zahinoor Ismail, Tomas Nikolai, Hana Markova, Katerina Cechova, Zuzana Nedelska, Jan Laczó, Meng Wang, Jakub Hort, Martin Vyhnalek

**Affiliations:** ^1^Memory Clinic, Department of Neurology, Second Faculty of Medicine, Charles University and Motol University Hospital, Prague, Czechia; ^2^International Clinical Research Center, St. Anne's University Hospital Brno, Brno, Czechia; ^3^Department of Psychiatry, Cumming School of Medicine, Calgary, AB, Canada; ^4^Department of Clinical Neurosciences, Cumming School of Medicine, Calgary, AB, Canada; ^5^Department of Community Health Sciences, Cumming School of Medicine, Calgary, AB, Canada; ^6^Hotchkiss Brain Institute and O'Brien Institute for Public Health, University of Calgary, Calgary, AB, Canada

**Keywords:** entorhinal cortex, hippocampus, mild behavioral impairment-checklist, mild cognitive impairment, neuropsychiatric symptoms, subjective cognitive decline, magnetic resonance imaging

The published article has now been updated from a Brief Research Report to an Original Research article, to better reflect its contents.

In the original article, there was also an error in an author's name as published. Instead of Jan Laczo it should be Jan Laczó.

The authors apologize for these errors and state that these do not change the scientific conclusions of the article in any way. The original article has been updated.

## Publisher's Note

All claims expressed in this article are solely those of the authors and do not necessarily represent those of their affiliated organizations, or those of the publisher, the editors and the reviewers. Any product that may be evaluated in this article, or claim that may be made by its manufacturer, is not guaranteed or endorsed by the publisher.

