# Supplementary material for: Mild Behavioral Impairment Is Associated With Atrophy of Entorhinal Cortex and Hippocampus in a Memory Clinic Cohort
Source: Front Aging Neurosci. 2021 May 24;13:643271. doi: 10.3389/fnagi.2021.643271 (PMC8180573; doi:10.3389/fnagi.2021.643271)
Supplement: Supplementary file 1 [file Table_1.DOCX]

Supplementary Material

# The associations between MBI-C and ROI excluding non-amnestic MCI participants

Table 1. Demographic and volumetric characteristics of the participants

| **Characteristics** | **All**  **n = 103**  **M ± SD** |
| --- | --- |
| **Age** | 70.00 ± 7.96 |
| **Female, n (%)** | 48 (47) |
| **MMSE, score** | 27.54 ± 2.51 |
| **Education** | 15.50 ± 3.54 |
| **RAVLT 1-5, score** | 40.38 **±** 14.31 |
| **RAVLT delayed recall, score** | 6.71 ± 4.91 |
| **LM delayed recall, score** ^c^ | 10.17 ± 6.28 |
| **WAIS-III Digit span, score** ^c^ | 14.75 ± 3.75 |
| **TMT A, time to completion (s)** ^c^ | 49.16 ± 27.35 |
| **TMT B, time to completion (s)** ^c^ | 134.62 ± 82.39 |
| **BNT-30, mistakes after a semantic cue** ^c^ | 3.90 ± 3.78 |
| **C-VF animals, score** ^c^ | 20.91 ± 6.82 |
| **P-VF, score** ^c^ | 42.29 ± 14.40 |
| **PST – colors, time to completion (s)** ^c^ | 37.50 ± 16.52 |
| **ROCF copy, score** ^c^ | 28.18 ± 5.67 |
| **ROCF recall, score** ^c^ | 12.37 ± 8.03 |
| **ERC, mm** | 3.02 ± 0.50 |
| **HV, mm^3^** | 3568.98 ± 634.42 |
| **ACC, mm** | 2.74 ± 0.20 |
| **PCC, mm** | 2.33 ± 0.19 |
| **OFC, mm** | 2.44 ± 0.20 |

Abbreviations: MMSE = Mini Mental State Examination; ERC = entorhinal cortex thickness; HV = hippocampal volume; ACC = anterior cingulate cortex thickness; PCC = posterior cingulate cortex thickness; OFC = orbitofrontal cortex thickness; M = mean; SD = standard deviation.

Table 2. MBI-C scores of the participants

| **MBI-C, score (range):** | **All**  **n = 103**  **M ± SD** |
| --- | --- |
| **Total (0-102)** | 4.54 ± 5.41 (0-27) |
| **Decreased motivation (0-18)** | 1.11 ± 1.75 (0-8) |
| **Affective dysregulation (0-18)** | 1.52 ± 2.08 (0-9) |
| **Impulse dyscontrol (0-36)** | 1.61 ± 2.31 (0-9) |
| **Social inappropriateness (0-15)** | 0.21 ± 0.52 (0-3) |
| **Abnormal perception/thought (0-15)** | 0.09 ± 0.35 (0-2) |

Abbreviations: MBI-C = Mild behavioral impairment checklist; M = mean; SD = standard deviation.

Table 3. Associations of cortical thickness and volume measures with MBI-C total and domain scores

|  | Spearman r_S_ , p value  adjusted for age, sex and education | | | | | | Spearman r_S_ , p value  adjusted for age, sex, education and MMSE | | | | | | |
| --- | --- | --- | --- | --- | --- | --- | --- | --- | --- | --- | --- | --- | --- |
|  | MBI-C total score | MBI-C  Motivation | MBI-C  Affectivity | MBI-C  Impulse dyscontrol | MBI-C  Social | MBI-C  Perception/thought | MBI-C total score | MBI-C  Motivation | MBI-C  Affectivity | MBI-C  Impulse dyscontrol | MBI-C  Social | MBI-C  Perception/thought |  |
| ERC† | -0.305, 0.002* | -0.123, 0.224 | -0.193, 0.055 | -0.391, <0.001* | -0.172, 0.086 | -0.182, 0.070 | -0.262, 0.009* | -0.072, 0.479 | -0.150, 0.137 | -0.391, <0.001* | -0.192, 0.057 | -0.195, 0.053 |  |
| HV†† | -0.241, 0.016 | -0.281, 0.005* | -0.064, 0.529 | -0.270, 0.007* | -0.159, 0.114 | 0.054, 0.591 | -0.180, 0.075 | -0.227, 0.024 | 0.004, 0.972 | -0.229, 0.023 | -0.188, 0.062 | 0.054, 0.591 |  |
| ACC† | 0.002, 0.985 | 0.085, 0.401 | -0.003, 0.975 | -0.043, 0.675 | -0.136, 0.179 | -0.097, 0.338 | -0.011, 0.915 | 0.075, 0.463 | -0.014, 0.887 | -0.053, 0.603 | -0.133, 0.188 | -0.097, 0.338 |  |
| PCC† | -0.010, 0.925 | 0.025, 0.803 | 0.036, 0.721 | -0.115, 0.253 | -0.092, 0.365 | -0.101, 0.316 | -0.004, 0.970 | -0.031, 0.758 | 0.042, 0.680 | -0.112, 0.268 | -0.093, 0.361 | -0.101, 0.316 |  |
| OFC† | -0.188, 0.061 | -0.163, 0.106 | -0.096, 0.342 | -0.203, 0.043 | -0.144, 0.153 | -0.093, 0.359 | -0.174, 0.086 | -0.148, 0.143 | -0.081, 0.425 | -0.192, 0.057 | -0.149, 0.142 | -0.095, 0.348 |  |

†cortical thickness (averaged between left and right hemisphere)

††volume (averaged between left and right hemisphere) adjusted for eTIV (proportion method)

*significant after applying Holm-Bonferroni correction for multiple comparisons; Abbreviations: MBI-C = mild behavioral impairment checklist; ERC = entorhinal cortex thickness; HV = hippocampal volume (eTIV); ACC = anterior cingulate cortex thickness; PCC = posterior cingulate cortex thickness; OFC = orbitofrontal cortex thickness.

# Associations between MBI-C and amygdala – an exploratory analysis

Table 4. Associations between MBI-C and amygdala

|  | Spearman r_S_ , p value  adjusted for age, sex and education  n=116 | | | | | | Spearman r_S_ , p value  adjusted for age, sex, education and MMSE  n=116 | | | | | | |
| --- | --- | --- | --- | --- | --- | --- | --- | --- | --- | --- | --- | --- | --- |
|  | MBI-C total score | MBI-C  Motivation | MBI-C  Affectivity | MBI-C  Impulse dyscontrol | MBI-C  Social | MBI-C  Perception/thought | MBI-C total score | MBI-C  Motivation | MBI-C  Affectivity | MBI-C  Impulse dyscontrol | MBI-C  Social | MBI-C  Perception/thought |  |
| AMG | 0,060, 0.530 | -0.089, 0.348 | -0.080, 0.400 | -0.063, 0.506 | 0.033, 0.730 | 0.053, 0.575 | 0,003, 0.977 | -0.035, 0.712 | -0.031, 0.745 | -0.013, 0.896 | 0.035, 0.714 | 0.056, 0.559 |  |

Abbreviations: AMG = amygdala volume (eTIV) averaged between left and right hemisphere, MBI-C = Mild behavioral impairment checklist; M = mean; SD = standard deviation.
